# The cost and cost trajectory of whole‐genome analysis guiding treatment of patients with advanced cancers

**DOI:** 10.1002/mgg3.281

**Published:** 2017-03-12

**Authors:** Deirdre Weymann, Janessa Laskin, Robyn Roscoe, Kasmintan A. Schrader, Stephen Chia, Stephen Yip, Winson Y. Cheung, Karen A. Gelmon, Aly Karsan, Daniel J. Renouf, Marco Marra, Dean A. Regier

**Affiliations:** ^1^ Canadian Centre for Applied Research in Cancer Control (ARCC)Cancer Control ResearchBC Cancer AgencyVancouverBritish ColumbiaCanada; ^2^ Division of Medical OncologyBC Cancer AgencyVancouverBritish ColumbiaCanada; ^3^ Canada's Michael Smith Genome Sciences CentreBC Cancer AgencyVancouverBritish ColumbiaCanada; ^4^ Department of Medical GeneticsFaculty of MedicineUniversity of British ColumbiaVancouverBritish ColumbiaCanada; ^5^ Department of Molecular OncologyBC Cancer AgencyVancouverBritish ColumbiaCanada; ^6^ Department of MedicineFaculty of MedicineUniversity of British ColumbiaVancouverBritish ColumbiaCanada; ^7^ Department of Pathology & Laboratory MedicineFaculty of MedicineUniversity of British ColumbiaVancouverBritish ColumbiaCanada; ^8^ Department of PathologyBC Cancer AgencyVancouverBritish ColumbiaCanada; ^9^ School of Population and Public HealthFaculty of MedicineUniversity of British ColumbiaVancouverBritish ColumbiaCanada

**Keywords:** Cost analysis, oncology, transcriptome sequencing, whole‐genome sequencing

## Abstract

**Background:**

Limited data exist on the real‐world costs of applying whole‐genome analysis (WGA) in a clinical setting. We estimated the costs of applying WGA to guide treatments for patients with advanced cancers and characterized how costs evolve over time.

**Methods:**

The setting is the British Columbia Cancer Agency Personalized OncoGenomics (POG) program in British Columbia, Canada. Cost data were obtained for patients who enrolled in the program from 2012 to 2015. We estimated mean WGA costs using bootstrapping. We applied time series analysis and produced 10‐year forecasts to determine when costs are expected to reach critical thresholds.

**Results:**

The mean cost of WGA over the study period was CDN$34,886 per patient (95% CI: $34,051, $35,721). Over time, WGA costs decreased, driven by a reduction in costs of sequencing. Yet, costs of other components of WGA increased. Forecasting showed WGA costs may not reach critical thresholds within the next 10 years.

**Conclusion:**

WGA costs decreased over the studied time horizon, but expenditures needed to realize WGA remain significant. Future research exploring costs and benefits of WGA‐guided cancer care are crucial to guide health policy.

## Introduction

Whole‐genome sequencing (WGS) or targeted gene panels represent a potential future standard of care in oncology (Kilpivaara and Aaltonen 2013; Manolio et al. 2013). Targeted gene panels identify mutations in a predetermined selection of genes, whereas WGS reveals the full spectrum of mutations by sequencing the whole genome of tumor and normal cells (Dienstmann et al. 2013; Laskin et al. 2015). While costs of applying WGS in a research setting have decreased over time, costs of subsequent bioinformatics analysis necessary to interpret sequence data remain substantial, a phenomenon commonly referred to as “the $1000 genome and the $100,000 analysis” (Mardis 2010; Caulfield et al. 2013; Wetterstrand 2016).

Costs of applying WGS and subsequent analysis (whole‐genome analysis, WGA) will vary across clinical and research settings. WGA will involve different workflow depending on whether it is applied for diagnostic, prognostic, or predictive purposes. Generally, the process of WGA involves a combination of sample preparation, WGS, and data processing including bioinformatics analysis, interpretation, and validation of genomic data (Ellis et al. 2012; Luheshi and Raza 2014; Plöthner et al. 2016). To date, little evidence on the real‐world cost, cost components, and cost trajectory of WGA have been published (Church 2006; Frank et al. 2013; CADTH, 2014; van Amerongen et al. 2016). Available estimates of WGA costs focus on expenditures related to procurement and running WGS platforms, often failing to describe costs of workflow or subsequent analysis (Frank et al. 2013). Researchers are beginning to report cost components of next‐generation sequencing (NGS) technologies and analysis using microcosting (Monroe et al. 2013; van Nimwegen et al. 2015; Plöthner et al. 2016; Sabatini et al. 2016; Costa et al. 2016), but there are currently no published costs of applying WGA to inform clinical care in oncology.

The British Columbia Cancer Agency (BCCA) Personalized OncoGenomics (POG) program was initiated in 2012 to apply a more comprehensive form of WGA. POG‐related WGA uses both WGS and transcriptome sequencing (RNA‐seq) to guide real‐time treatment planning for patients with treatment‐resistant cancers (Laskin et al. 2015). Throughout this article, we use the term WGA to represent POG‐related WGA. POG began as a feasibility study to build a program that integrates genomic data into clinical decision making. In addition to potentially generating clinically actionable findings for cancer patients, POG is establishing processes for incorporating WGA into routine cancer care. As WGA becomes increasingly applied in clinical care, comprehensive cost analyses are necessary to guide health system planning.

Our study objective is to estimate the average costs of WGA and to characterize how total costs and cost components evolve over time. Our setting is the BCCA POG program, located in British Columbia, Canada. We estimate mean WGA costs and apply time series analysis to understand how costs change over time. We then forecast when costs of WGA are expected to reach critical thresholds.

## Materials and Methods

### Ethical compliance

The University of British Columbia BCCA Research Ethics Board approved this study.

### Data

We obtained patient‐level WGA cost data from the BCCA Genome Sciences Centre from July 2012 to December 2015. Patients recruited to POG had advanced cancers involving different primary tumor sites, including cancers of unknown primaries (Laskin et al. 2015). Those who consented to participate in the program underwent WGA (Figure S1), beginning with an initial biopsy from their tumor site (usually a metastatic site), a peripheral blood sample, and retrieval of their archival diagnostic tissue sample, used to study changes in tumor biology. If the biopsy was successful, WGS and Ion Ampliseq‐focused cancer panel sequencing were applied to all samples. Panel sequencing served as an orthogonal validation tool of WGS data. We excluded patients whose biopsy samples contained insufficient tumor material for WGS (13.3% of cases). Blood samples, representing “normal” DNA controls, were sequenced to ~40‐fold redundant coverage and tumor samples were sequenced to exceed ~80‐fold redundant coverage. RNA‐seq (~200 million reads per patient tumor sample) was applied to biopsy samples for transcriptome analyses to identify and evaluate dysregulated gene expression, to confirm genomic alterations, and to identify candidate drug targets. Bioinformatics analysis integrated potentially actionable genetic variants with gene expression patterns using a purpose‐built data analysis pipeline to (1) determine the genes and pathways that were critical to individual malignancies, (2) identify candidate therapeutic susceptibilities, and (3) guide treatment planning with available systemic therapies, including clinical trials and/or off‐label therapeutic agents. The analyzed data and a list of potentially actionable or informative features were then compiled into a standardized report format, which was reviewed by a multidisciplinary tumor board and provided to clinicians.

When possible, sequencing results were further validated using immunohistochemistry, fluorescence in situ hybridization, or targeted sequencing. Validation was applied more frequently following POG program changes in July 2014, marking the transition from Version 1.0 to Version 2.0 of the program. Changes also included upgrading HiSeq 2500 platforms with “1 TB upgrade”, processing more POG patients, discontinuing sequencing in “rapid run” mode (relatively costly method for sequencing fewer samples at high speed), and discontinuing sequence analysis of diagnostic tissue samples. In Version 2.0, costs of reagents and materials purchased from American suppliers increased owing to changes in the American exchange rate (Wangaryattawanich et al. 2015).

The BCCA Genome Sciences Centre recorded patient‐level expenditures for major components of WGA, including costs of (1) biopsy and sample processing (pathology, radiology, staining, sectioning, and blood draws), (2) panel sequencing (including bioinformatics and report generation), (3) WGS and RNA‐seq, (4) bioinformatics analysis related to WGS and RNA‐seq (computation and alignments to identify candidate driver mutations, genes, and pathways, tool development to facilitate this process, software upgrades, interpretation, and drug‐based report generation), (5) validation (verification experiments and confirmatory testing), (6) PET scans (performed in approximately 20% of POG cases to assess changes in disease status), and (7) other fixed program costs (e.g., salaries for full‐time staff to prepare and sequence samples, apply bioinformatics analysis, and interpret results as well as equipment costs, including sequencing platform upgrades). We aggregated costs for each patient to obtain an estimate of total WGA costs. We estimated average monthly sequencing and WGA costs based on patients’ month of biopsy. We linearly interpolated missing monthly data (7.1% of observations). Sensitivity analysis involving cubic interpolation resulted in little difference in interpolated values and had no material effect on model conclusions. All costs are reported in 2015 Canadian dollars.

### Statistical analysis

We estimated mean WGA costs across patients from July 2012 to December 2015 and between Version 1.0 and 2.0 of the program. We applied nonparametric bootstrapping to simulate sampling distributions of total WGA costs and costs for each component of WGA (Barber and Thompson 2000). We used two‐sided *t*‐tests and Mann–Whitney *U* nonparametric tests to determine whether WGA costs significantly differed across program versions (Mann and Whitney 1947). We identified statistical significance using a threshold of *P* < 0.05.

To examine changes in sequencing costs over time, we specified autoregressive integrated moving average models with explanatory variables (Appendix S1). Explanatory variables included intercepts, linear time trends, dummy variables denoting level changes (changes in mean costs after a particular time point), and interaction terms for trend changes (changes in the rate at which costs change over time). When appropriate, we modelled changes in the variability of costs. We identified possible level changes, trend changes, and changes in variability (termed structural breaks) based on prior knowledge of POG program events and visual inspection. We tested hypothesized structural breakpoints using Chow tests and *F*‐tests (Chow 1960).

Using coefficient estimates from final time series models, we produced 10‐year dynamic forecasts to determine when costs are expected to reach critical thresholds, $1000, $3000, or $5000 per patient. These price targets were chosen in proximity to the frequently cited “$1000 genome,” which is considered low enough to make genome sequencing routine (Church 2006; Pareek et al. 2011; Wright 2011). We examined three forecast scenarios for each model. For WGA costs, the first scenario involved a baseline forecast using only the final model specification, the second scenario allowed for an additional 1% decrease in costs in each month following December 2017, and the third scenario allowed for a 50% reduction in the rate at which costs changed from December 2017 to December 2019. For WGS and RNA‐seq costs, the first scenario involved a baseline forecast using only the final model specification, the second scenario allowed for an additional 1% decrease in costs in each month following December 2015, and the third scenario allowed for a 50% reduction in the rate at which costs changed after December 2015.

Scenarios highlight the sensitivity of our forecasts to various assumptions about the future of genome sequencing and WGA costs. We allowed for a variety of forecast shocks to determine potential net effects of technological advances, automated pipelines, data storage bottlenecks, and changes in interpretation demands. We performed all analyses using Stata version 13 (StataCorp L, 2013).

## Results

### Total cost and cost components

POG enrolled 301 patients during the study period. Of these individuals, 84 participated in Version 1.0 of the program and 217 participated in Version 2.0. Enrollment in POG increased over the period (Figure S2). Patients enrolled in POG had varying cancer diagnoses (Table 1). Overall, the most common primary tumor sites among POG patients were breast cancer and gastrointestinal cancers, mirroring the relative prevalence of these cancer types encountered at the BCCA (BC Cancer Statistics ‐ Facts and Figures, 2016).

**Table 1:** Primary tumor sites of patients enrolled in POG, July 2012 to December 2015

Primary tumor site	Overall (*n* = 301)		Version 1.0 (*n* = 84)		Version 2.0 (*n* = 217)	
	*n*	%	*n*	%	*n*	%
Breast	77	26	28	33	49	23
Gastrointestinal (including pancreas)	64	21	8	10	56	26
Sarcoma	29	10	5	6	24	11
Other	29	10	4	5	25	12
Lung	29	10	9	11	20	9
Gynecologic	26	9	8	10	18	8
Head and Neck	18	6	9	11	9	4
Unknown	13	4	4	5	9	4
Skin	6	2	3	4	3	1
Hematologic/hematolymphoid	5	2	2	2	3	1
Adrenal	3	1	2	2	1	0
Peritoneal	2	1	2	2	0	0

Differences in frequency distributions of primary tumor sites across versions are statistically significant (*P* < 0.05).

Table 2 summarizes cost and cost components of WGA. On average, WGA cost $34,886 per patient (95% CI: $34,051, $35,721). WGS and RNA‐seq drove the majority of costs, with a mean cost per patient of $19,400 (95% CI: $18,404, $20,395). Mean bioinformatics costs were $5143 per patient (95% CI: $5241, $5406) and an estimated ~50% of these costs corresponded to interpretation and reporting. Remaining bioinformatics costs corresponded to standard analysis through existing applications and pipelines. Mean WGA costs decreased by 12% across POG versions, from $38,042 (95% CI: $35,488, $40,597) in Version 1.0 (2012–2014) to $33,665 (95% CI: $33,168, $34,161) in Version 2.0 (2014–2015), driven by a 47% decrease in mean WGS and RNA‐seq costs and a 44% decrease in panel sequencing costs. The proportion of WGS and RNA‐seq costs attributed to RNA‐seq increased from 14% in Version 1.0 to 20% in Version 2.0, following discontinuing WGS of matched diagnostic tissue samples. Mean costs of bioinformatics, validation, and other fixed costs increased across POG versions.

**Table 2:** Summary of WGA costs per patient by POG program version, July 2012 to December 2015

Cost element	Overall				Version 1.0				Version 2.0			
	Median cost per patient	Mean cost per patient	Standard error of mean	95% CI of mean	Median cost per patient	Mean cost per patient	Standard error of mean	95% CI of mean	Median cost per patient	Mean cost per patient	Standard error of mean	95% CI of mean
Total WGA costs^a^^,^ b	$33,132	$34,886	$426	$34,051, $35,721	$36,426	$38,042	$1303	$35,488, $40,597	$32,957	$33,664	$253	$33,168, $34,161
Biopsy and sample processing^c^	$521	$588	$19	$550, $625	$627	$625	$26	$574, $676	$477	$574	$24	$527, $621
Panel sequencing^a^, ^b^, ^c^	$1613	$1530	$44	$1443, $1617	$2245	$2239	$60	$2122, $2356	$1600	$1255	$42	$1172, $1338
WGS and RNA‐seq^a^, ^b^, ^c^	$15,195	$19,400	$508	$18,404, $20,395	$28,283	$29,500	$1196	$27,156, $31,845	$14,751	$15,490	$200	$15,098, $15,881
Bioinformatics^a^^,^ c	$5143	$5323	$42	$5241, $5406	$5143	$5157	$104	$4953, $5362	$5574	$5388	$41	$5307, $5468
Validation^a^, ^b^, ^c^	$0	$476	$71	$337, $616	$0	$2	$2	−$2, $5	$0	$660	$92	$479, $841
PET scans	$0	$225	$29	$168, $283	$0	$174	$55	$67, $281	$0	$245	$34	$179, $311
Other fixed costs^a^, ^b^, ^c^	$10,133	$7344	$264	$6827, $7860	$0	$346	$207	−$60, $752	$10,133	$10,053	$65	$9925, $10,180

a Differences in mean costs across POG Version 1.0 and Version 2.0 are statistically significant (*P* < 0.05) – Satterthwaite's approximation for unequal variances.

b Differences in mean costs across POG Version 1.0 and Version 2.0 are statistically significant (*P* < 0.05) – bootstrapped standard errors.

c Differences in distribution of costs across POG Version 1.0 and Version 2.0 are statistically significant (*P* < 0.05).

### Changes in costs over time

**Figure 1:**
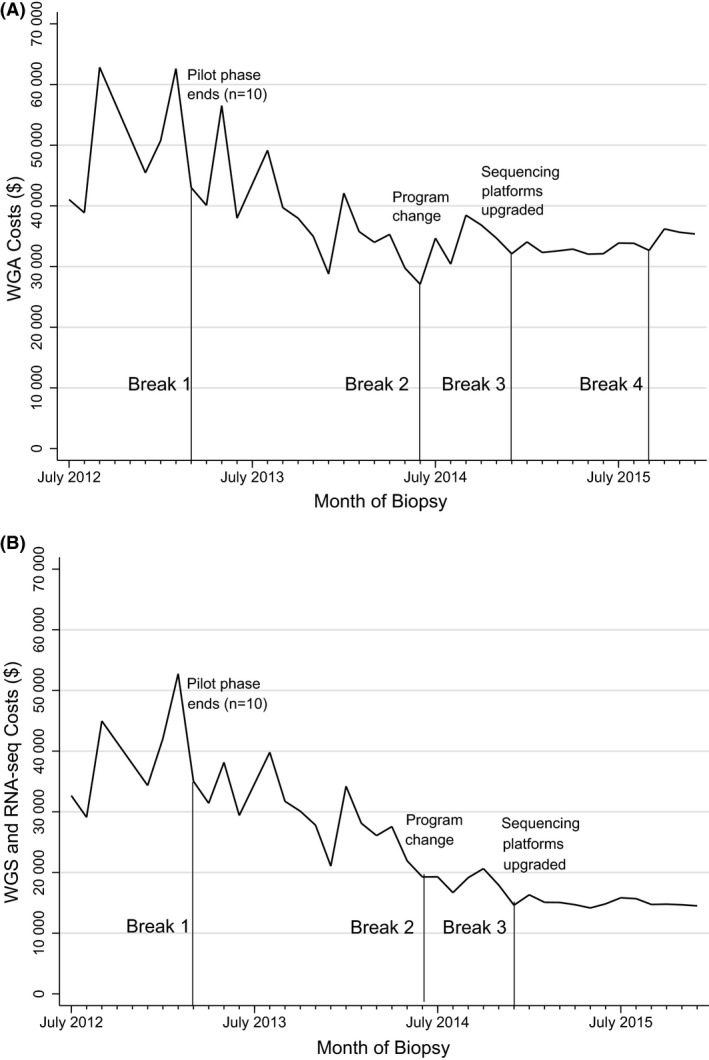
Trends in (A) WGA costs and (B) WGS and RNA‐seq costs from July 2012 to December 2015.

Figure 1A and B depicts changes in average WGA costs and WGS and RNA‐seq costs over time, including significant structural breakpoints. Results from time series models are in Table 3. Mean WGA costs increased by $1156 per month (95% CI: $387, $1925) from July 2012 until the first breakpoint in March 2013, when the pilot phase of patients receiving WGA ended and enrollment increased. From March 2013 to June 2014, mean WGA costs decreased by $1286 per month (95% CI: −$1549, −$1023). After the second breakpoint in June 2014, when the POG program began transitioning from Version 1.0 to 2.0, mean costs declined by $184 per month (95% CI: −$274, −$94) until December 2015. WGA costs became less variable after the third breakpoint in December 2014, when sequencing platforms were upgraded. Mean WGA costs increased at the fourth breakpoint in July 2015.

**Table 3:** Results from ARIMAX models of total WGA costs and WGS and RNA‐seq costs

Outcome	WGA costs		WGS and RNA‐seq costs	
	Coefficient	SE	Coefficient	SE
Intercept	45,755.68^a^	1651.84	31,568.27^a^	1687.22
Trend	1156.05^a^	392.45	1701.73^a^	480.34
Break 1 – level change	14,003.96^a^	2317.70	9999.63^a^	1938.23
Break 1 – trend change	−2441.80^a^	350.79	−2361.03^a^	379.84
Break 2 – level change	−20,620.30^a^	2739.83	−19,405.49^a^	2073.07
Break 2 – trend change	1101.79^a^	140.25	467.98^a^	130.25
Break 4 – level change	3706.03^a^	689.30		
Multiplicative heteroskedasticity				
Break 3 – level change	−3.16^a^	0.69	−3.80^a^	1.32
Intercept	16.77^a^	0.36	16.23^a^	0.34
*n*	42		42	
ARMA disturbances	AR (1–7)		AR (1 and 7) MA (2)	

Augmented Dickey–Fuller and Dickey–Fuller generalized least squares tests indicated that our residual series were stationary after accounting for statistically significant structural breaks (Elliott et al. 1992; Fuller 2009). All specified models fully accounted for autocorrelation. After modelling significant breaks in variance, models showed no evidence of autoregressive conditional heteroskedasticity.

a Statistically significant coefficient estimates (*P* < 0.05).

**Figure 2:**
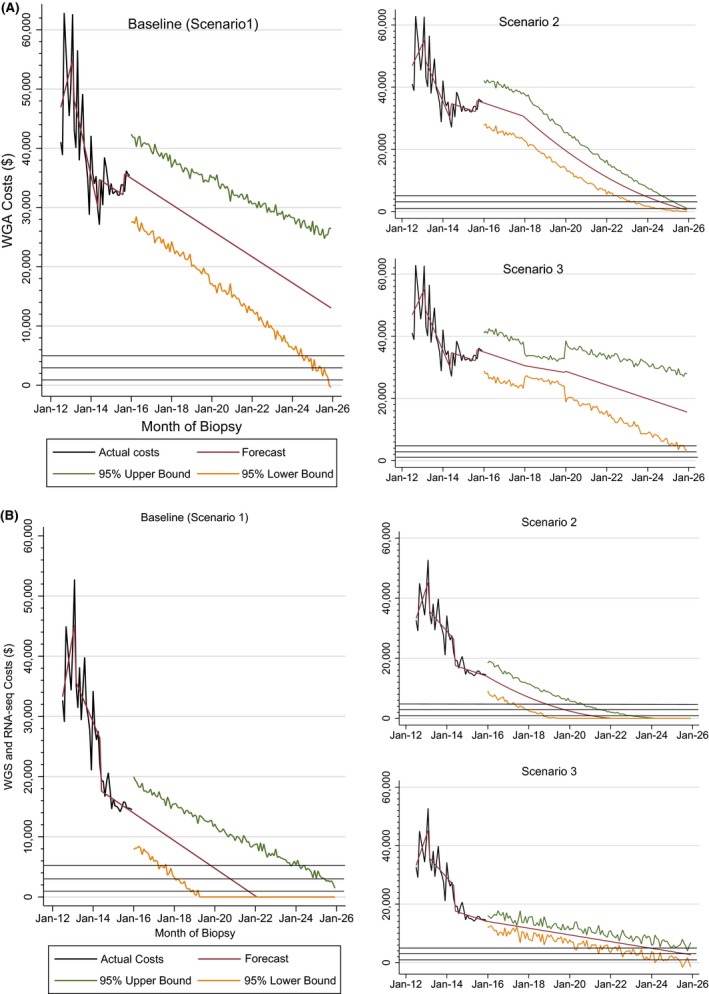
Ten‐year forecasts of (A) total WGA costs and (B) WGS and RNA‐seq costs.

Figure 2A illustrates 10‐year forecast scenarios for WGA costs. Under our baseline scenario, we do not expect mean WGA costs to reach $5000 per patient within the next 10 years. The lower bound of our 95% forecast interval does include $1000 in June 2025. In our second scenario, accounting for an additional 1% decrease in WGA costs in each month following December 2017, forecasted mean costs reach $5000 per patient in December 2023, $3000 in September 2024, and $1000 in September 2025. In our third scenario, allowing for a 50% reduction in the rate at which WGA costs changed from December 2017 to December 2019, we do not expect mean WGA costs to reach $5000 per patient within the next decade.

We also examined the trajectory of WGS and RNA‐seq costs. From July 2012 to March 2013, average WGS and RNA‐seq costs increased by $1702 per month (95% CI: $760, $2643). After the first breakpoint, in March 2013, mean costs decreased by $659 per month (95% CI: −$958, −$361) until June 2014. After the second breakpoint, in June 2014, costs declined by $191 per month (95% CI: −$269, −$114) until December 2015. WGS and RNA‐seq costs became less variable after the third breakpoint in December 2014.

Figure 2B illustrates 10‐year forecast scenarios for WGS and RNA‐seq costs. Under our baseline scenario, we expect mean WGS and RNA‐seq costs will reach $5000 per patient by December 2019, $3000 by November 2020, and $1000 by September 2021. Under our second scenario, accounting for an additional 1% decrease in WGS and RNA‐seq costs in each month following December 2017, forecasted mean costs reach $5000 per patient by November 2018, $3000 by October 2019, and $1000 by January 2021. In our third scenario, allowing for a 50% reduction in the rate at which costs changed after December 2015, we expect mean costs to reach $5000 per patient by December 2023 and $3000 by September 2025, but we do not expect costs to reach $1000 per patient in the next 10 years.

## Discussion

In this study, we analyzed total cost and cost components of POG‐related WGA from July 2012 to December 2015. We found, on average, WGA cost CDN$34,886 per patient over the time period. WGA costs decreased over time, driven by a reduction in WGS and RNA‐seq costs, which fell from CDN$29,500 per patient in Version 1.0 to CDN$15,490 in Version 2.0 of POG. In a research setting, WGS costs fell from CDN$6988 per genome to CDN$4570 per genome over the same period (Wetterstrand 2016). Excluding bioinformatics costs and other cost components, the cost trajectory of WGS and RNA‐seq observed in our study mirrors the WGS cost trajectory reported in a research setting. Our higher mean costs likely stem from differences in implementation, which involved applying both WGS and RNA‐seq, sequencing multiple samples, and achieving a relatively high coverage rate (>80‐fold rather than 30‐fold coverage).

Although WGS costs are declining, changes are partially offset by increasing costs of bioinformatics analysis, validation costs, and other fixed program costs. Increasing fixed costs reflect sequencing platform upgrades and the need to employ full‐time staff to process more patients’ data, which was not necessary in the first version of the POG program. Increasing bioinformatics costs present additional challenges. As costs of data generation decline, long‐term data storage costs may not decline. It could soon be cheaper to regenerate data from DNA samples than to store sequence data (Batley and Edwards 2009; Sboner et al. 2011; Stephens et al. 2015). Changes may be offset by technological advances, including the ongoing development of more efficient sequencing platforms (e.g., HiSeq X platforms) and nanopore technologies, increasing throughput, an expanding knowledge base for interpreting sequence data, and superior automation of bioinformatics and interpretation, for instance the use of in silico probes to identify common actionable mutations. Further exploration of the trajectory of nonsequencing costs is necessary to guide health policy and planning.

Proponents of precision medicine claim that sequencing genomes at a cost of $1000 each will soon be feasible (Church 2006; Pareek et al. 2011; Wright 2011). Forecasts confirm that applying WGS and RNA‐seq at a cost of $1000 per patient may be achievable in as few as 6 years. We project it will take substantially longer before comprehensive WGA reaches a similarly low threshold within a clinical setting. Applying NGS to the complexity of human cancers generates a vast amount of complex data, which is challenging to interpret and incorporate into treatment planning for individual patients. Targeted gene panels have been proposed as a cost‐efficient alternative to WGA and other NGS technologies, and some emerging evidence supports their cost‐effectiveness relative to existing standards of care (Metzker 2010; Xue et al. 2014; Gallego et al. 2015). Research suggests that gene panels are less applicable to clinical scenarios involving extreme heterogeneity (Xue et al. 2014). In these scenarios, panels identify fewer clinically actionable results than other NGS technologies (Laskin et al. 2015). Our analysis shows targeted gene panels cost approximately $1500 per patient, on average. This cost is not inconsequential if gene panels fail to identify optimal treatment options for patients. Furthermore, WGA may generate cost savings as it is more likely to identify potential resistance mechanisms to new, costly therapeutic agents. If used as a first‐line testing strategy, WGA may also result in earlier diagnoses and avoidance of ineffective treatments. Future research exploring the trade‐off between costs and clinical utility of these technologies is warranted.

### Study limitations

Our study is not without limitations. Our data on the cost trajectory of WGA are limited to 3 years, the majority of which involved building a new program, and 10‐year forecasts involve considerable extrapolation. We assume WGA costs will behave similarly in the future as they did in our sample and long‐term forecasts are subject to significant uncertainty. By forecasting several possible scenarios, we gain insight into the potential effects of future shocks on WGA costs and the sensitivity of our models to such departures. We also determine the maximum cost to apply WGA in 10 years with no further breaks in the trajectory of costs.

Another limitation of our study corresponds to our setting. Our data record costs of applying a comprehensive form of WGA, involving WGS, RNA‐seq, and panel sequencing, to identify treatment options for patients with incurable cancers, some with highly resistant disease. This application of WGA may be particularly complex when compared to other settings and our estimates of mean WGA costs may be relatively high (Tsiplova et al. 2016). Despite this potential limitation, POG's integrated approach allows for a more thorough exploration of the oncogenesis of an individual's cancer and is comparable to comprehensive WGA approaches in other international programs, such as the Michigan Oncology Sequencing Project or Genome England's 100,000 Genomes project (Roychowdhury et al. 2011; Caulfield et al. 2015). Furthermore, we believe it is likely that this patient population will be the first to obtain access to WGA when it is incorporated on a wider scale, given the elevated costs of WGA. We believe that our findings concerning changes in total costs and cost components of WGA over time are likely representative of changes in WGA costs within other clinical settings and can be used to inform health system planning.

## Conclusion

Costs of WGA are decreasing, but mean expenditures needed to realize WGA and guide treatments for patients with advanced cancers remain high. We found some cost elements are increasing and there are a number of factors to consider going forward. Reaching critical thresholds, particularly within a clinical setting, will take time and incorporating WGA on a wider scale will require significant monetary investment. Despite these costs, WGA offers many potential benefits and future research exploring the trade‐off between costs and benefits of WGA‐guided cancer care is essential to inform health policy and planning.

## Conflict of Interest

The authors have no conflicts of interest to declare.

## Disclaimer

The views expressed in the submitted article are the authors’ own and not an official position of the funder.

## Supporting information


**Figure S1.** Process for POG‐related whole‐genome analysis.
**Appendix S1.** Detailed description of time series analysis.
**Figure S2.** Number of patients enrolled in POG from July 2012 to December 2015.
