# Supplementary material for: The cost and cost trajectory of whole‐genome analysis guiding treatment of patients with advanced cancers
Source: Mol Genet Genomic Med. 2017 Mar 12;5(3):251–60. doi: 10.1002/mgg3.281 (PMC5441418; doi:10.1002/mgg3.281)
Supplement: Supplementary file 1 — Figure S1. Process for POG‐related whole‐genome analysis. Appendix S1. Detailed description of time series analysis. Figure S2. Number of patients enrolled in POG from July 2012 to December 2015. [file MGG3-5-251-s001.doc]

**APPENDIX A - Process for POG-related whole-genome analysis**

**APPENDIX B – Detailed description of time-series analysis**

We estimated autoregressive integrated moving average models with explanatory variables (ARIMAX). In addition to allowing for differencing of series to remove stochastic trends and achieve stationarity, ARIMAX models enabled us to model correlations in costs over time using autoregressive (AR) and moving average (MA) terms.[^1^](#_ENREF_1) AR terms allow current costs to depend on lagged costs and MA terms allow current costs to depend on prior random shocks. These shocks pertain to random fluctuations around the main trend in a time-series.

Explanatory variables included intercepts, linear time trends, dummy variables denoting level changes, and interaction terms denoting trend changes. Intercepts estimate the mean monthly cost at the beginning of the period and linear time trends estimate the rate at which monthly costs are changing. Level changes (changes in mean costs after a particular point in time) or trend changes (changes in the rate at which costs change over time) can occur after a shift in the time-series, known as a structural break. Structural breaks can also result in variance changes (changes in the variability of costs). We identified possible structural breaks in costs based on prior knowledge of POG program events and visual inspection. We tested for hypothesized structural breaks using Chow tests and F-tests.[^2^](#_ENREF_2) When appropriate, we modelled variance breaks by accounting for multiplicative heteroskedasticity within conditional variance equations.[^1^](#_ENREF_1)

We applied augmented Dickey-Fuller and Dickey-Fuller generalized least squares unit root tests to verify whether residual series were stationary and we used a combination of residual diagnostics, Breusch-Godfrey tests, and Ljung-Box Q tests to determine whether our models fully accounted for autocorrelation.[^3-8^](#_ENREF_3) To establish whether our models showed evidence of autoregressive conditional heteroskedasticity (ARCH), we applied Engle’s Lagrange multiplier test.[^9^](#_ENREF_9)

Using coefficient estimates from our final ARIMAX models, we produced ten-year dynamic forecasts of WGA costs and WGS and RNA-seq costs. We estimated statistical precision of all forecasts using Monte Carlo simulation methods accounting for both parameter estimate uncertainty and uncertainty surrounding error terms based on static forecast residuals. Using these estimates, we calculated 95% prediction intervals for our forecasts.

**APPENDIX C**

**Supplemental Figure 1: Number of patients enrolled in POG from July 2012 to December 2015**

**REFERENCES**

**1.** Greene W. *Econometric Analysis.* 7 ed. New York: Prentice Hall; 2008.

**2.** Chow GC. Tests of equality between sets of coefficients in two linear regressions. *Econometrica: Journal of the Econometric Society.* 1960:591-605.

**3.** Fuller WA. *Introduction to statistical time series.* Vol 428: John Wiley & Sons; 2009.

**4.** Elliott G, Rothenberg TJ, Stock JH. Efficient tests for an autoregressive unit root. National Bureau of Economic Research Cambridge, Mass., USA; 1992.

**5.** Breusch TS. Testing for autocorrelation in dynamic linear models*. *Australian Economic Papers.* 1978;17(31):334-355.

**6.** Godfrey LG. Testing against general autoregressive and moving average error models when the regressors include lagged dependent variables. *Econometrica: Journal of the Econometric Society.* 1978:1293-1301.

**7.** Box GE, Pierce DA. Distribution of residual autocorrelations in autoregressive-integrated moving average time series models. *Journal of the American statistical Association.* 1970;65(332):1509-1526.

**8.** Ljung GM, Box GE. On a measure of lack of fit in time series models. *Biometrika.* 1978;65(2):297-303.

**9.** Engle RF. Autoregressive conditional heteroscedasticity with estimates of the variance of United Kingdom inflation. *Econometrica: Journal of the Econometric Society.* 1982:987-1007.
